# NFKB1 and Cancer: Friend or Foe?

**DOI:** 10.3390/cells7090133

**Published:** 2018-09-07

**Authors:** Julia Concetti, Caroline L. Wilson

**Affiliations:** Newcastle Fibrosis Research Group, Institute of Cellular Medicine, Newcastle University, Newcastle upon Tyne, Tyne and Wear NE2 4HH, UK; j.concetti2@ncl.ac.uk

**Keywords:** NF-κB, NFKB1, p105/p50, Bcl-3, cancer, inflammation, apoptosis

## Abstract

Current evidence strongly suggests that aberrant activation of the NF-κB signalling pathway is associated with carcinogenesis. A number of key cellular processes are governed by the effectors of this pathway, including immune responses and apoptosis, both crucial in the development of cancer. Therefore, it is not surprising that dysregulated and chronic NF-κB signalling can have a profound impact on cellular homeostasis. Here we discuss NFKB1 (p105/p50), one of the five subunits of NF-κB, widely implicated in carcinogenesis, in some cases driving cancer progression and in others acting as a tumour-suppressor. The complexity of the role of this subunit lies in the multiple dimeric combination possibilities as well as the different interacting co-factors, which dictate whether gene transcription is activated or repressed, in a cell and organ-specific manner. This review highlights the multiple roles of NFKB1 in the development and progression of different cancers, and the considerations to make when attempting to manipulate NF-κB as a potential cancer therapy.

## 1. Introduction

One of the emerging questions in cancer biology is: “How are inflammation and dysregulated immune responses linked to cancer?” It is now widely accepted that chronic inflammation and infection represent major risk factors for certain cancers. Increasing evidence suggests that the cellular effectors and signalling pathways involved in these pathological contexts play a substantial role in defining both the development and progression of cancer. Among those, NF-κB transcription factors play a fundamental role in the regulation of immune responses, apoptosis, and cell survival, and hence, are implicated as central mediators of cancer initiation and progression. Examples include strong links to inflammation-driven cancers such as colitis-associated cancer, MALT (mucosal-associated lymphoid tissue) lymphoma, and HBV (hepatitis B virus) or HCV (Hepatitis C virus) driven HCC (hepatocellular carcinoma) [1]. Whilst mutations in the upstream activators and individual subunits of NF-κB are rare, it is far more likely that increased and even consistent NF-κB activation is promoted by inflammatory stimuli, the source of which is likely to vary widely from underlying inflammatory conditions (i.e., viral hepatitis and EBV (Epstein-Barr virus), cellular senescence and senescence associated secretory proteins and immune activation by malignant cells) [2,3]. Indeed, dynamic cross-talk between malignant and immune cells further promote this activation to support tumour growth and survival [4]. NF-κB activation leads to the NF-κB-dependent transcription of genes coding for inflammatory cytokines, cell-cycle modulators, survival signals, and growth and angiogenic factors, all key drivers in a tumour-promoting environment. Due to the vast array of proteins under the control of NF-κB, attempts to inhibit this process with blanket inhibitors of upstream effectors such as IκB Kinase (IKK) inhibitors have had limited success, compounded by the off-target effects [5]. Balance between NF-κB activation and control is lost during pathological conditions such as chronic inflammation and cancer. To understand and more importantly regain control over this aberrant NF-κB activation, there is a need to understand more about de-activating NF-κB at both a cell-specific and molecular level (i.e., the NF-κB subunits).

## 2. NF-κB Subunits

**Figure 1:**
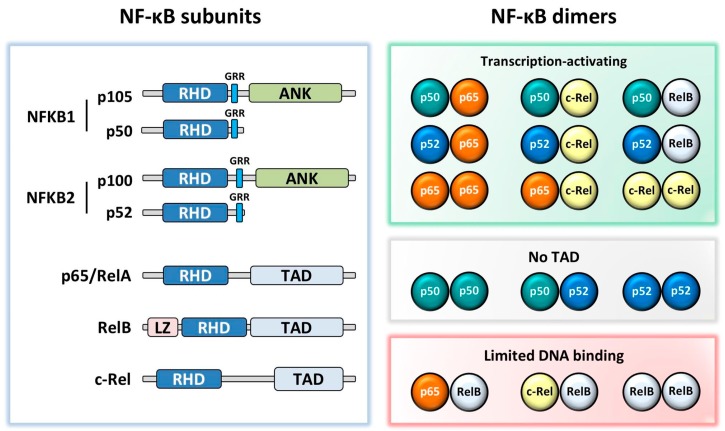
NF-κB subunit structures and dimeric combinations. All subunits contain a Rel homology domain (RHD). NFKB1 and NFKB2 have a glycine rich region (GRR), followed by an Ankyrin repeat domain (ANK) in the precursors p105 and p100. p65, RelB, and c-Rel contain a transactivation domain (TAD), with RelB additionally containing a leucine zipper (LZ) motif. While most dimers activate transcription, p50:p50, p50:p52, and p52:p52 dimers lack a TAD, and therefore repress transcription in the absence of co-activating factors and p65:RelB, c-Rel:RelB, and RelB:RelB dimers are thought to have limited DNA binding [8,9,10].

NF-κB consists of five subunits: p65 (RelA), RelB, c-Rel, p50 (NFKB1), and p52 (NFKB2). Unlike the other subunits, *NFKB1* and *NFKB2* are synthesized as precursors (p105 and p100) which are proteolytically cleaved to p50 and p52, respectively. The NF-κB subunits, which form homo- and hetero-dimers, are kept inactive in the cytoplasm by inhibitor of κB proteins (IκB); phosphorylation and degradation of IκB through activation of the NF-κB signalling pathway leads to the translocation of NF-κB dimers into the nucleus. These bind to target gene κB promoter sites via their Rel homology domain. Whilst the most well-known and commonly studied NF-κB dimer is p50:p65, NF-κB exists in several other dimer combinations with different and important functional outputs [6]. p65, RelB, and c-Rel all contain a transcriptional activation domain (TAD) enabling them to drive gene transcription in their homodimer form. However, this TAD is absent in p50 and p52, therefore they must either form a heterodimer with p65, c-Rel or RelB, or recruit co-activators to drive gene transcription, and when bound to DNA as homodimers they are generally thought to be transcriptional repressors (Figure 1) [7].

## 3. NFKB1 Processing

The *NFKB*1 gene, which comprises 24 exons, is located on chromosome 4q24 [11]. Upon stimuli, p105 is phosphorylated in its C-terminal domain containing an ankyrin repeat (similar to that of the IκB inhibitory proteins), followed by partial degradation in an ubiquitin-proteasome dependent manner. The degradation of the inhibitory C-terminal region subsequently leads to p50 activation [12]. Distinct from the role of p50, p105 also plays an important role in the stabilisation of pre-formed NF-κB dimers. In mice where p105 serine residues 927 and 932 are mutated to alanine, there is impaired p105 processing and as a result p50:p65 release and activity is inhibited [13,14]. Aside from its direct role in the control of NF-κB activation, p105 also plays an additional role in stabilising Tpl2 kinase (tumour progression locus 2). p105 inhibits Tpl2-mediated activation of the MEK/ERK (mitogen-activated protein kinase kinase/extracellular regulated kinase) pathway and this inhibition is abrogated following IKK-induced proteolysis of p105 [15]. The role of p105 in the regulation of this pathway has been extensively reviewed [16].

## 4. Co-Factor Recruitment

p50 homodimers repress inflammation by competing with activating NF-κB dimers and preventing them from binding to κB sites on the promoters of target genes, further strengthened by the recruitment of co-repressors. Histone deacetylases (HDAC) repress gene transcription by removing acetyl groups from histones, often leading to chromatin condensation and transcriptional repression. The p50:p50:HDAC1 complex is able to repress multiple inflammatory genes, including *GM-CSF*, *CCL2*, *CXCL-10*, and *MMP-13* [17]. We have recently identified the site of p50:HDAC1 interaction and shown that mutagenesis of this binding site on p50 prevents HDAC1 binding with p50 homodimers. As a result of this loss of P50:HDAC1 interaction, we observed increased expression of the inflammatory genes *CXCL1*, *CXCL2*, and *IL-6* both basally and in response to stimulus (in press).

The IκB family protein B cell lymphoma 3 (Bcl-3) plays a dual role in p50 transcriptional regulation. As a co-activator it can bind p50 via ankyrin repeats and help drive gene transcription when complexed with p50 homodimers, and is also able to recruit other transcriptional regulators including STAT1, AP-1, c-Jun, and MMP-13 c-fos [18]. However, Bcl-3 can also stabilize the p50 homodimer by masking ubiquitination sites and preventing removal from gene promoters [19]. Therefore, Bcl-3 can also function as a co-repressor of gene transcription by preventing transcriptionally active NF-κB dimers from binding to these gene promoters and driving transcription [19]. Indeed, following LPS (lipopolysaccharide) stimulation of macrophages, p50:p50:Bcl-3 complexes negatively regulate *TNFα* (tumour necrosis factor α), *IL-1α*, and *IL-1β* expression, while activating anti-inflammatory *IL-10* gene transcription. In this context Bcl-3 enhances p50 homodimer-dependent TNFα repression, as p50 is also able to repress TNFα in the absence of Bcl-3, though to a lesser extent [20]. However, it is highly unlikely that these complexes are as simple as this; it is far more realistic to view these known protein interactions as parts of the jigsaw when in reality they are likely to form large multimeric complexes consisting of numerous co-factors. In support of this, both HDAC1 and Bcl-3 have also been found in nuclear complexes together in LPS stimulated cells [20].

Other factors known to interact with and modulate p50 homodimer activity include C/EBP (CCAAT enhancer binding protein) which has been shown to displace HDAC1 and HDAC3 bound to p50 homodimers, abrogating anti-apoptotic gene repression and contributing to aberrant p50 activity in acute myeloid leukemia [21]. Also, the co-activator p300 regulated by PARP (Poly (ADP-ribose) polymerase) is necessary for p50 transcriptional activation in primary lung fibroblasts following TNFα and LPS stimulation [22].

The dual ability of p50 to both activate and repress gene transcription instigates a complex relationship between this NF-κB subunit and cancer, with much controversy as to its role played in carcinogenesis. Given the complex and diverse nature of cancer as a disease, it is not surprising that the function of NFKB1 can differ substantially in a cell, organ, and cancer specific manner. In this review, we will discuss how NFKB1 is implicated in carcinogenesis, acting both as a pro- and anti-tumorigenic transcription factor.

## 5. NFKB1 Tumour Suppressor

p50 homodimers are primarily considered to repress gene transcription, dampen inflammatory responses, and abrogate anti-apoptotic signalling [17,23,24]. Chronic inflammation and the evasion of apoptosis are hallmarks of cancer [25], hence it is no surprise that there is an abundance of emerging evidence supporting a role for p50 homodimers as tumour-suppressors. KPC1 (KIP1 ubiquitination-promoting complex 1) was recently identified as the ubiquitin ligase that mediates the processing of p105 to p50. Kravtsova-Ivantsiv and colleagues [26] showed that KPC1 overexpression in a mouse xenograft tumour model derived from either the glioblastoma cell line (U87-MG cells) or the human breast cancer cell line (MDA-MB 231 cells) inhibits tumour growth via increased p50 generation. This was further supported by p50 overexpression that also reduced the growth of both tumours. Interestingly, p65 expression was significantly decreased in KPC1 and p50 overexpressing tumours, and an increase in the formation of p50 homodimers over p50-p65 heterodimers was confirmed by EMSA. The increase in p50 homodimer formation resulted in downregulated genes including *HMGI-C*, *lin-28 homolog A*, *IL-6, IL-6R* and *VEGFA* and an upregulation of tumour suppressor genes including *PTEN* (Phosphatase and tensin homolog). Moreover, in support of this, in a human context both p50 and KPC1 expression was significantly decreased in human head, neck, and glioblastoma tumours compared to normal tissue, and p50 expression decreased in breast cancer tumours [26]. Together their findings strongly suggest that KPC1, and hence p50, inhibit the growth of various tumours, likely via inhibition of p50:p65-mediated pro-tumorigenic gene transcription.

### 5.1. Liver Cancer

The role of NF-κB in liver cancer has been extensively studied and its first role in liver homeostasis highlighted in p65 deficient mice which suffer embryonic lethality due to TNFα-induced hepatocyte death during development [27]. Contrasting and cell-specific effects of IKK deletion in liver epithelial versus myeloid cells highlight the complexity of its role at different stages of HCC development. Knockdown of IKKβ in hepatocytes leads to increased liver cancer, whereas knockdown in myeloid cells results in significantly decreased hepatocarcinogenesis [28]. These studies of course merely highlight the implications of inhibiting the entire canonical NF-κB pathway which researchers are beginning to appreciate is far more complex and requires further refinement if we are to manipulate it in a more specific and productive way, including a more in-depth understanding of the epigenetic regulation and post-translational modifications of the individual subunits.

We have previously provided evidence that NFKB1, acting through p50 homodimers, is a suppressor of neutrophil-driven hepatocellular carcinoma (HCC). We previously demonstrated using a model of diethylnitrosamine (DEN)-induced HCC that *Nfkb1^−/−^* mice exhibited accelerated HCC and significantly increased tumour numbers compared to wild type mice. This was mediated in part by p50 homodimers complexed with HDAC1 repressing the hepatic expression of the neutrophil chemokines S100A9, CXCL1, and CXCL2. Treatment with a neutrophil-depleting antibody (anti-Ly6G) reduced tumour growth considerably in *Nfkb1^−/−^* mice. Furthermore, we identified that the phosphorylation of Ser342 is critical for p50 homodimer assembly, and that *Nfkb1^−/−^* mice with a serine to alanine mutation at position 342(S342A) display increased neutrophil numbers, neutrophil chemokine expression, and increased tumour burden in the DEN HCC mouse model. Combined this data confirmed that p50 homodimers in complex with HDAC1 play an important role in preventing liver inflammation and tumorigenesis by repressing neutrophil recruitment and as a result, neutrophil tumour-promoting effects. Interestingly, in a non-experimentally-induced context, aged 20 month old *Nfkb1^−/−^* mice also develop spontaneous chronic liver disease and liver cancer characterised by dysplastic nodules, increased tumour incidence, features of steatohepatitis and fibrosis [23].

In HCC patients where ~90% of cancers develop on a background of aberrant liver inflammation, we have also confirmed that an increase in neutrophil to lymphocyte ratio is associated with a poorer prognosis and worsened survival outcome [29]. Furthermore, mRNA, miRNA, and methylation profiling of 337 HCC patients identified *NFKB1* and the MAPK (mitogen-activated protein kinase) pathway as important players in HCC progression. Here, *NFKB1* inhibition was mediated via the miRNA let-7a at all stages of HCC, resulting in aberrant target gene regulation including *Gadd45β* and dysregulated cell proliferation and apoptosis. In addition, NFKB1 was differentially methylated in stage II and III HCC, but not stage IV [30]. The miRNA silencing of *NFKB1* and its differential methylation could therefore be a potential mediator of HCC development; however, further research is needed in order to elucidate the specific role of p50 in different stages of HCC including liver inflammation and disease, and cancer initiation and progression. Whilst contrasting studies exist including correlating p50 expression with early recurrence of HCC and Akt phosphorylation, p65 expression was not assessed here [31]. Increased p50 and Bcl-3 co-expression in tumours compared to adjacent tissue has also been described [32]. However, these studies simply describe p50 expression levels and fail to address functionality and mechanism.

### 5.2. Gastric Cancer

Drivers of gastric cancer (GC) include poor diet, obesity, and chronic infection. One of the main infection-driven gastric cancers is caused by *Helicobacter pylori. Helicobacter Pylori* produces a number of virulence factors able to activate the NF-κB pathway, which in turn lead to the recruitment of inflammatory cells and the production of inflammatory mediators that help drive tumorigenesis [33]. In conjunction with liver cancer studies the complexity of NF-κB activation in gastric tumorigenesis has also been highlighted using IKK cell-specific knockouts. IKKβ^−/−^ in the gastric epithelial cell compartment leads to accelerated development of dysplasia, whereas knockout in only myeloid cells inhibited progression to gastric atrophy and dysplasia [34]. However, also in correlation with liver cancer development, mice lacking *Nfkb1* develop spontaneous invasive GC. Here the *Nfkb1^−/−^* mice display characteristics of gastric cancer in humans including T cell and NK cell infiltration, increased pro-inflammatory gene expression including *IL-1β*, *IL-6*, *TNFα* and *osteopontin*, and the metalloproteinases *MMP-7*, *MMP-9* and *MMP-13*. Mechanistically, this is explained by aberrant JAK-STAT (Janus kinase-Signal transducer and activator of transcription) signalling via increased pro-inflammatory mediators, STAT1 expression and increased expression of the immune checkpoint inhibitor PD-L1 [35]. Importantly, in this model *Nfkb1* expression was required in both the epithelial and immune cell compartments in order to prevent GC. The findings of this study are consistent with human GC patient data linking *NFKB1* gene polymorphisms, including rs28362491, with reduced p105/p50 expression and GC development [36,37]. In contrast, both NFKB1 and p65 have been shown at higher levels in GC cell lines and primary human GC tumours when compared to normal gastric epithelial cells which correlated with poor survival in patients [38]. Mechanistically, it was revealed that the NFKB1 targeting miRNA miR-508-3p was downregulated in GC cells, suggesting a tumour-suppressive function for this miRNA [38]. This reiterates the potentiality that p50:p65 dimers are pro-tumorigenic drivers as opposed to p50 homodimers in this context.

### 5.3. Lung Cancer

Aside from the suppressive effects of p50 homodimers, p105 itself can also exert a tumour-suppressive function. A recent study highlighted a role for p105 as a tumour suppressor via stabilisation of Tpl2. In a urethane-induced lung cancer mouse model which re-enacts human lung cancers associated with tobacco smoking, both *Nfkb1^−/−^* and *Tpl2^−/−^* mice showed a significant increase in lung tumour size and number when compared to wild type mice. Following the observation that Tpl2 expression was also decreased in a *NFKB1^−/−^* human lung cancer cell line, Fan Sun and colleagues [39] demonstrated that re-expressing p105, and not p50, restored Tpl2 levels in these cells, resulting in reduced tumour growth. Contrastingly, Tpl2 knockdown increased lung tumour cell growth. This study highlights the importance of p105 stabilisation of Tpl2 in suppressing lung carcinogenesis [39]. Analysis of human non-small-cell lung carcinoma (NSCLC) tumours graded from stage 1 to 3A revealed that p105 stromal and epithelial tumour cell expression was a positive prognostic indicator of disease-specific survival [40]. However, it has also recently been shown that co-expression of p65 and phosphorylated p105 is associated with poor prognosis in NSCLC, whereas expression of p65 or phosphorylated p105 alone was not associated with this [41]. Again, this strongly suggests that it is p50:p65 dimer activity and not p50 homodimer activity that is detrimental in NSCLC.

### 5.4. Haematological Malignancies

mRNA expression analysis has revealed that *NFKB1* is downregulated in multiple human haematological malignancies, including T and B-cell lymphoma and acute myeloid leukemia, while p65 expression is upregulated. DNA alkylation damage leads to increased lymphomas in *Nfkb1^−/−^* mice when compared with wild type mice. Mice heterozygous for *Nfkb1* exhibit an intermediary phenotype whilst maintaining p50 function, indicating the haploinsufficient nature of *Nfkb1* as a tumour suppressor [42]. Interestingly, p50 phosphorylation by ATR (ataxia telangiectasia mutated and Rad3-related kinase) at serine 329 ensures the elimination of replication-associated DNA-damaged cells and is necessary for genome maintenance. Phosphorylation of p50 at this site during the S phase of the cell cycle inhibits its activity and DNA binding, downregulating the expression of the anti-apoptotic protein *Bcl-xL* sensitizing cells to DNA strand breaks. Transfection of *Nfkb1^−/−^* MEFs (mouse embryonic fibroblasts) with S329A mutant p50 prevents p50 phosphorylation and inhibition, and blocks the observed decrease in *Bcl-xL* expression during S phase [43]. Similarly, it has also been shown that S329 p50 phosphorylation is involved in promoting apoptosis in cells that have undergone O6-methylguanine (O6-MeG) DNA lesions, by preventing the expression of anti-apoptotic genes [24]. Therefore, p50 may play a protective role in preventing the survival of DNA-damaged cells that have the potential to become cancerous. In support of this, it was shown by EMSA that in adult T-cell leukemia (ATL) patients, NF-κB-DNA complexes consist of both p50:p65 heterodimers and p50 homodimers, whereas in healthy patients they are predominantly p50 homodimers. Moreover, only p50:p65 dimers, and not p50 homodimers, were able to activate the transcription of *IL-2Rα* in ATL patients, demonstrating that constitutive activation of p50:p65 is a major driver of ATL [44]. These studies highlight that aberrant p50:p65, rather than p50:p50 signalling could be implicated in haematological malignancies where DNA-damaged cells have evaded apoptosis.

## 6. NFKB1 Tumour Promoter

In contrast to p50:p50 homodimers, an increase in DNA bound p50:p65 heterodimers leads to increased expression of NF-κB regulated genes including pro-inflammatory (*IL-1β*, *TNFα*, *CXCL*1), anti-apoptotic (*Bcl-xL*, *Bcl-2*, *Gadd45β*) and proliferative (*IL-6*, *GM-CSF*) to name a few. Therefore, it is not surprising that NFKB1 acts as a tumour promoter in this context. In addition, there are also a number of cancers including lymphomas and colorectal cancer where recruitment of co-activators will actually promote the expression of a similar array of tumour-promoting genes.

### 6.1. Bcl-3-Associated Cancers

The aforementioned Bcl-3 complexed with p50 homodimers is associated with tumorigenesis in several malignancies [45]. Notably, classical Hodgkin/Reed-Sternberg (HRS), anaplastic large cell lymphoma (ALCL) and T cell lymphomas, exhibit constitutive p50 homodimer activity associated with Bcl-3, verified by EMSA and IP shift [45]. Additionally, increased p50 and Bcl-3 expression in HRS and ALCL cell lines are mainly in the nuclear fractions verified by co-immunoprecipitation and is associated with increased expression of the anti-apoptotic genes *Bcl-xL*, *c-IAP2*, and *TRAF-1* in the HRS cell lines [45].

In accordance with this, several other studies confirm that increased tumour expression of p50 (and not p65) correlates with an increased expression of Bcl-3. For instance, mouse skin papillomas and squamous cell carcinomas (SCC) display increased p50 and p52 expression compared to normal epithelial tissue from the middle stages of cancer development, while p65 levels remained unchanged. This correlates with increased Bcl-3 expression in late stage skin papillomas and SCC, suggesting a role for the p50:p50:Bcl-3 complex in tumour promotion [46]. Other examples of co-expression in malignant tissue include HPV16 (human papilloma virus 16) positive oral cancers, nasopharyngeal carcinoma and breast cancer [47,48,49]. Together these findings suggest that p50 homodimers in complex with Bcl-3 may play an important role in carcinogenesis, likely via the upregulation of NF-κB target genes. Importantly, to verify where p50:p50:Bcl3 complexes truly function as tumour promoters in-depth molecular analysis such as EMSA, nuclear localisation, IP and analysis of other NF-κB subunits should be included.

### 6.2. Breast and Gynaecological Cancers

Studies involving breast and gynaecological cancers suggest an association with increased *NFKB1* expression and nuclear localisation. Increased p50 DNA binding and to a lesser extent p65 binding has been observed in a subset of high-risk estrogen receptor positive breast cancers [50]. Also, knock-down of *NFKB1* in the inflammatory breast cancer cell line (SUM-149) suggests that *NFKB1* expression positively regulates Rho C expression and cell motility, which may contribute to the metastatic phenotype of inflammatory breast cancer [51]. Overexpression of p105 along with p100 in cervical carcinoma-derived keratinocytes expressing the HPV16 oncoproteins E7 or E6, show localisation is predominantly cytoplasmic when E7 is expressed, and nuclear in the case of E6; therefore, these viral oncoproteins may actually dictate the subcellular localisation of p105 and p100 and hence activity of p105 and p100 [52]. Contrasting results exist in cervical cancer describing both increased nuclear p50 and p65 correlating with poor tumour grade and larger tumour size, and increased expression of predominantly p50 homodimers with no observed increase in p65 [53,54]. Whereas in ovarian cancer it is both p50 and p65 that are increased when compared to borderline and benign tumours, indicating the involvement of p50:p65 heterodimers rather than p50 homodimers [55].

However, caution must be applied when thinking about mechanism and the cell-specific implications of NF-κB. Whilst there are many studies focused on subunit expression in tumours, specifically that of p50 and p65, as suggested earlier, epithelial versus immune cell NF-κB activation could have very different outputs. For instance, p50 and not p65 is overexpressed in tumour-associated macrophages from human ovarian cancer. As a result, p50:p50 homodimers repress the activation signals required for M1 polarisation leading to defective IL-12 production. When *Nfkb1^−/−^* mice are implanted with murine fibrosarcoma there is no observed defect in M1 cytokine production and tumour growth is significantly reduced. The *Nfkb1^−/−^* mice also show enhanced CXCL-10 chemokine expression and hence CD4+ and CD8+ T cell infiltration that help combat tumour growth in this model [56]. Thus, highlighting the importance of dissecting cell-specific roles of NFKB1 if we are to attempt to manipulate its function as a potential future therapy for inflammation and cancer.

### 6.3. Colorectal and Pancreatic Cancers

Similar to the previously mentioned study [56], it has also been recently described that p50 homodimers impair macrophage M1 polarisation, driving the development of colorectal cancer [57]. In this study, *Nfkb1^−/−^* mice displayed fewer colorectal tumours and increased expression of IL-12 and CXCL-10, supporting the idea that p50 homodimers repress these genes in macrophages, orchestrating their pro-tumorigenic phenotype [57]. BAG-1 (Bcl-2 associated athanogene) is an anti-apoptotic protein highly expressed in pre-malignant and colon cancer tissue. The interaction between BAG-1 and p50 homodimers has been demonstrated in both colorectal epithelial cells and in the colorectal carcinoma cell line HCT116 with knockdown of either protein leading to cell death. This complex is present at both the *EGFR* and the *COX2* gene (PTGS2) promoters. Furthermore, the complex is able to differentially regulate gene expression by suppressing transcription of *EGFR* and promoting *COX-2* transcription, both known to promote colon cancer [58].

Increased p50 signalling is also implicated in pancreatic carcinogenesis. Annexin A2 (ANXA2), is a calcium-dependent phospholipid binding protein involved in the progression and metastasis of a number of tumours and is shown to interact and translocate to the nucleus in a complex with p50 [59]. Increased NF-κB activity is observed in both resting and TNFα stimulated pancreatic cancer cells (Mia-Paca2). Furthermore, TNFα treatment of HeLa cells expressing ANXA2 leads to the induction of several NF-κB target genes linked to anti-apoptotic signalling and drug resistance in cancer, including *GM-CSF*, *IL-1β*, *IL-6* and *Gadd45β*. This suggests a link between increased ANXA2 expression and p50 interaction leading to increased anti-apoptotic gene expression and drug resistance in pancreatic cancer cells [59]. However, in a mouse pancreatic tumour model, increased tumour growth was observed when pancreatic cancer cells were co-injected with p50^−/−^ compared to wild type PSC (pancreatic stellate cells), suggesting a potential role for PSC p50 in pancreatic prevention [60]. Thus, NFKB1 plays very contrasting roles in the development of colon and pancreatic cancers, and further research is needed to elucidate its cellular and context-dependent function.

### 6.4. Haematological Malignancies

In an Eμ-TCL1 mouse model of chronic lymphocytic leukemia, the incidence of leukemia is significantly lower in mice lacking *Nfkb1*, with CD19/CD5^+^ B cell numbers decreased despite no difference in overall survival. *Nfkb1^+/−^* TCL1 mice still show a significant reduction in leukemia incidence, demonstrating that even a partial reduction in *Nfkb1* can impede disease development [61]. It is known that intrinsic B cell defects are characteristic of mice deficient in *Nfkb1*, including proliferative defects and impairment in antibody class-switching [62]. Therefore, NFKB1 could be directly linked to B cell proliferation in chronic lymphocytic leukemia, characterised by an abnormally increased level of B cells in the lymph nodes, bone marrow, and blood [63].

Diffuse large B-cell lymphoma (DLBCL) has two distinct molecular subtypes: germinal centre B cell like (GCB) and activated B cell like (ABC). ShRNA silencing of either p105 or p100 in two lymphoma cell lines identified a set of distinctly regulated genes that were later confirmed in primary DLBCL samples. Pathway analysis identified patterns of predominantly p105 signalling associated with the GCB lymphoma subtype, whereas p100 was associated with ABC potentially as a consequence of mutations in upstream activators of either the canonical or non-canonical pathway respectively [64]. Moreover, in a study conducted on 465 patients, p50 activation in ABC DLBCL was associated with poorer survival, despite significant correlations between p50 nuclear expression and downregulation of Bcl-2, p53, phospho-AKT, CXCR4 and Myc. The poor outcome in p50 positive patients was attributed to immune dysregulation including immune suppression by TIM-3 and TNFα upregulation. A similar correlation was found in GCB DLBCL patients with wild-type TP53, however p50 expression in GBC DLBCL patients with TP53 mutants was associated with a significantly improved outcome, as well as reduced expression of Bcl-2, Myc and p53 [65].

**Figure 2:**
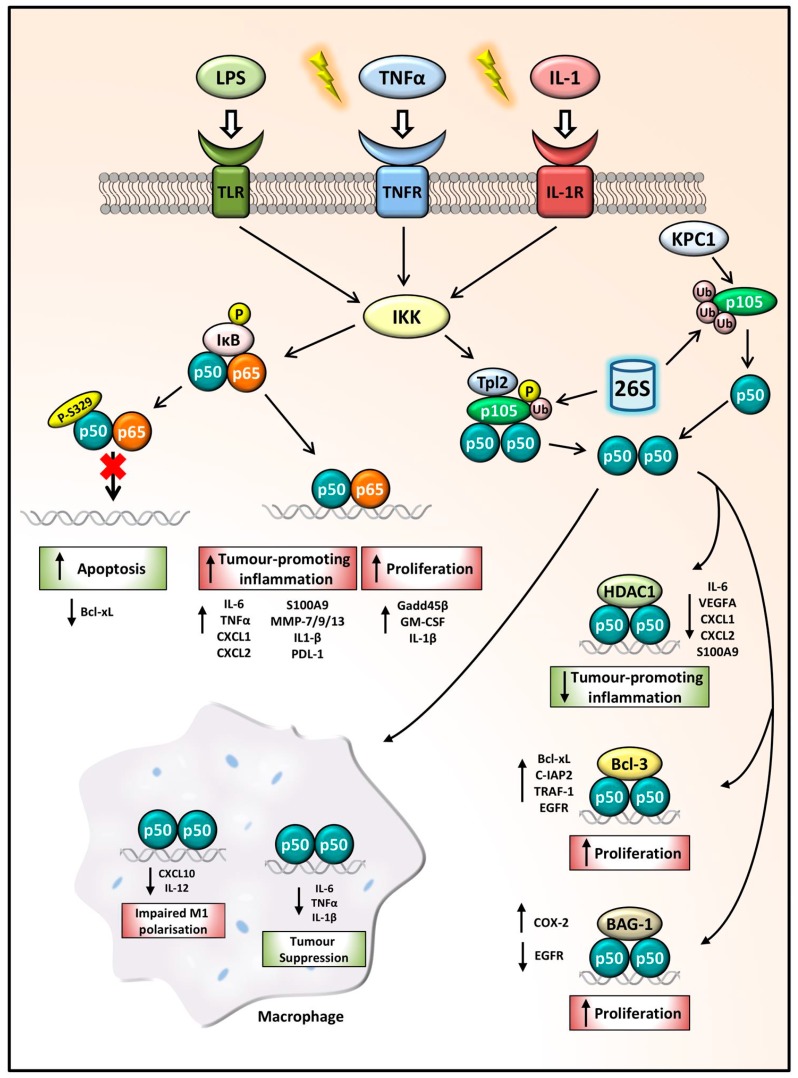
NFKB1 p105/p50 regulation of gene expression in cancer. Following inflammatory stimulation, IKK functions as the key activator of the NFKB1 signalling pathway. Phosphorylation and ubiquitination of IκB releases p50:p65 dimers which can drive the transcription of tumour-promoting inflammatory genes and anti-apoptotic and proliferative genes. p50 phosphorylation at the S329 site impairs p50:p65 dimer binding to DNA, decreasing *Bcl-xL* expression leading to increased apoptosis, hindering cancer progression. p105 phosphorylation and ubiquitination leads to its processing by the 26S proteasome, releasing p50 homodimers. The p50:p50:HDAC1 complex represses tumour-promoting inflammatory gene expression thus acting as a tumour-suppressor complex, while p50 homodimers in complex with Bcl-3 or BAG-1 can drive proliferation. In macrophages, aberrant p50 homodimer repression of *CXCL10* and *IL-12* leads to impaired M1 polarisation. Whilst p50:p50 repression of pro-inflammatory and proliferative mediators can also promote tumour suppression.

The role of p50 therefore differs substantially depending on its dimerization partner and the co-factors involved, as well as the cell type and cancer type, whereby gene expression can either be repressed or activated, hindering or driving tumorigenesis (summarised in Figure 2).

## 7. NFKB1 Polymorphisms in Cancer

*NFKB1* polymorphisms have been largely associated with immune defects including autoimmunity [66,67]. However, there are also links to either increased or decreased cancer risk in several different populations and cancer types (Table 1). Polymorphisms in the *NFKB1* gene have been suggested as risk factors for the development of several cancers including liver, gastric and ovarian cancers. These small changes in the *NFKB1* nucleotide sequence may impact protein function and expression through transcriptional regulation, since the polymorphisms are often located in potential regulatory regions such as introns and promoter sites. These polymorphisms have been predominantly studied in Asian populations, where it may confer a selective advantage for other cellular processes or diseases. However, fewer studies regarding *NFKB1* polymorphisms and cancer risk have been conducted in Western populations. Hence, further studies are required in order to extrapolate the cancer risk association to a wider population. In addition, there is a need to understand the functional effects of the different polymorphisms not only to give us more information about the physiological processes governed by this gene, but also to provide us with more information on how to harness its function in a variety of inflammatory scenarios and disease settings. Indeed, restoring the advantageous *NFKB1* nucleotide sequence through gene editing may also represent an effective therapeutic intervention in future years.

## 8. Potential Interventions

Drugs that stabilize or promote p50 homodimers have the potential to act as powerful anti-inflammatory and anti-cancer therapies. However, we still know very little about what dictates dimer partner choice and co-factor recruitment. Ongoing research focused on uncovering the specific amino acids responsible for the regulation and stabilization of the NF-κB subunits will prove vital in understanding their regulatory roles and post-translational modifications for future drug development. These include in vivo work exploring the use of peptide mimetics to strengthen p50 homodimer:DNA complexes to prevent inflammation which have huge potential as anti-cancer therapeutics [85].

## 9. Conclusions and Future Perspectives

The overarching role played by NFKB1 in carcinogenesis still remains incompletely understood. Contrasting mechanisms exist depending on the cancer and cell type, and uncertainty as to which dimerization partners and co-factors are involved form the basis of this conundrum. For these reasons more research is required to better delineate the specific role of NFKB1 in each cancer type. The emergence of data from genetic mouse models such as the S340A defective in p50 homodimers and the cell-specific knockouts *Nfkb1^−/−^* floxed mice will provide more functional insights into its role in a cancer-specific context. Overall, strong evidence suggests a protective and anti-tumorigenic role for p50 homodimers, particularly in HCC for example, whereas p50:p65 heterodimers are generally considered to be pro-inflammatory and pro-tumorigenic particularly where there is aberrant NF-κB activation. Therapeutic interventions targeting upstream activators of NF-κB directly may prove unsafe and non-specific causing toxicity to normal cells and tissues. However, manipulating NF-κB subunit dimerization, degradation or co-factor interaction may represent a safer, more targeted therapy in the treatment of cancer. For example, the emerging potential therapies aimed at stabilising p50 homodimer co-repressor complexes and target gene promoter binding are a promising approach. A number of *NFKB1* polymorphisms are now associated with cancer progression; therefore, further elucidating the functional effect of these polymorphisms may also bring us one step closer to the development of powerful anti-inflammatory and anti-cancer therapies in the future.

## Figures and Tables

**Table 1:** *NFKB1* SNPs (Single Nucleotide Polymorphisms) associated with cancer. *NFKB1* polymorphisms in different c-94ancer types are associated with either an advantageous or disadvantageous prognosis in different population cohorts.

SNP	Cancer Type	Sequence; Location; Consequence (If Known)	Prognostic	Cohort	Reference
**rs28362491**	Colorectal	ATTG del; -94 promoter region; reduces promoter activity and nuclear protein binding ability, decreasing NFKB1 expression	Disadvantageous	Swedish; Malaysian	[68,69]
	Gastric		Disadvantageous	Japanese; Chinese	[36,37]
	Liver		Disadvantageous	Taiwanese	[70]
	Bladder		Disadvantageous	Chinese	[71]
	Esophageal		Disadvantageous	Northern Indian	[72]
	Thyroid		Disadvantageous	Chinese	[73]
	Melanoma		Disadvantageous	Brazilian, Swedish	[74,75]
	Nasopharyngeal		Disadvantageous	Chinese	[76]
	Oral		Disadvantageous	Taiwanese	[77]
	Prostate		Advantageous	Canadian	[78]
	Ovarian		Advantageous	Greek	[55]
	Cervical		Advantageous	Chinese, Indian	[79,80]
**rs230496**	Liver	AG and GG genotypes	Disadvantageous	Chinese	[81]
**rs230525**	Liver	GA genotypes	Disadvantageous	Chinese	[81]
**rs230530**	Liver	AA genotypes	Disadvantageous	Chinese	[81]
**rs78696119**	Gastric	GG genotypes; increased inflammatory cell infiltration, diffuse type gastric cancer and cancer progression	Disadvantageous	Japanese	[36]
**rs72696119**	Gastric	GG and del/del genotypes; -449 in 5′ UTR region; muscle layer tumour invasion and lymph node metastasis	Disadvantageous	Japanese	[36]
**rs4648068**	Gastric	GG genotype; intron 12; increased incorporation of the H3K9me1 and H3K4me1 histones, increased chemomodification, enhanced transcriptional activity, cell proliferation and motility	Disadvantageous	Chinese	[37,82]
**rs230510**	Gastric	A genotype; intronic region	Advantageous	Japanese, US	[83]
	Ovarian	T genotype; intron 12	Advantageous	Chinese	[84]
**rs230521**	Ovarian	G genotype; intron 4	Advantageous	Chinese	[84]

## Abbreviations

ALCL	anaplastic large-cell lymphoma
BAG-1	BCL-2 associated athanogene
Bcl-3	B-cell lymphoma 3
C/EBP	CCAAT enhancer-binding protein
EBV	Epstein–Barr virus
EGFR	epidermal growth factor receptor
EMSA	electrophoresis mobility shift assay
ERK	extracellular regulated kinase
HCC	hepatocellular carcinoma
HDAC1	histone deacetylase 1
HPV16	human papilloma virus 16
IFN	interferon
IKK	IκB kinase
IL	interleukin
IκB	inhibitor of kappa light polypeptide gene enhancer in B-cells
JAK-STAT	Janus kinase-Signal transducer and activator of transcription
KPC1	Kip1 ubiquitination-promoting complex 1
LPS	lipopolysaccharide
MEK	mitogen-activated protein kinase kinase
NFKB1	nuclear factor of kappa light polypeptide gene enhancer in B-cells 1
NF-κB	nuclear factor of kappa light polypeptide gene enhancer in B-cells
NSCLC	non-small-cell lung carcinoma
PD-L1	programmed death-ligand 1
PTEN	phosphatase and tensin homolog
SCCHN	squamous cell carcinoma of the head and neck
STAT	signal transducer and activator of transcription
TAD	transcriptional activation domain
TIM-3	T-cell immunoglobulin and mucin-domain containing-3
TNFα	tumour necrosis factor α
TPL-2	tumour progression locus 2
